# Family Functioning and Executive Functions among ADHD Children

**DOI:** 10.1192/j.eurpsy.2023.628

**Published:** 2023-07-19

**Authors:** W. Walenista, B. Izydorczyk, K. Sitnik-Warchulska, I. Markevych, C. Baumbach, Y. Mysak, M. Szwed, M. Lipowska

**Affiliations:** ^1^ Institute of Applied Psychology; ^2^Institute of Psychology, Jagiellonian University, Kraków, Poland

## Abstract

**Introduction:**

ADHD is one of the most frequently diagnosed neurodevelopmental disorders and affects the daily functioning of families raising children with this condition. Among the symptoms typical for ADHD, low effectiveness of executive functions can determine the quality of family life.

**Objectives:**

This study aimed to specify whether family communication and satisfaction as reported by a parent are predictors of a child’s executive functioning quality and whether ADHD severity lies on the pathway between the two. Moreover, the child’s sex effect was checked.

**Methods:**

The study included 200 Polish participants (nGirls = 56) from the NeuroSmog project aged 10-13 diagnosed with ADHD according to the ICD-11. Stanford-Binet 5 Intelligence Scale, PU1 Cognitive Diagnosis Battery, Conners 3 ADHD Diagnosis Questionnaire, and the FACES IV Questionnaire were used to derive needed information. Structural equation modelling (SEM) was applied to test the hypotheses.

**Results:**

The quality of family communication and satisfaction did not predict the child’s executive functioning of ADHD children and ADHD severity did not play a mediating role. No differences by sex were observed. We only found a significant effect between IQ and executive functioning level in the general sample (standardized β = X, p = Y) and in girls (-0,24, 0,007).

**Conclusions:**

These results contrast with previous studies from other cultural contexts that have shown the existence of the hypothesized interrelations. Further research should confirm or refute these observations.

**Disclosure of Interest:**

W. Walenista Grant / Research support from: the TEAM-NET programme of the Foundation for Polish Science, co-financed from EU resources, obtained from the European Regional Development Fund under the Smart Growth Operational Programme, B. Izydorczyk Grant / Research support from: the TEAM-NET programme of the Foundation for Polish Science, co-financed from EU resources, obtained from the European Regional Development Fund under the Smart Growth Operational Programme, K. Sitnik-Warchulska Grant / Research support from: the TEAM-NET programme of the Foundation for Polish Science, co-financed from EU resources, obtained from the European Regional Development Fund under the Smart Growth Operational Programme, I. Markevych Grant / Research support from: the TEAM-NET programme of the Foundation for Polish Science, co-financed from EU resources, obtained from the European Regional Development Fund under the Smart Growth Operational Programme, C. Baumbach Grant / Research support from: the TEAM-NET programme of the Foundation for Polish Science, co-financed from EU resources, obtained from the European Regional Development Fund under the Smart Growth Operational Programme, Y. Mysak Grant / Research support from: the TEAM-NET programme of the Foundation for Polish Science, co-financed from EU resources, obtained from the European Regional Development Fund under the Smart Growth Operational Programme, M. Szwed Grant / Research support from: the TEAM-NET programme of the Foundation for Polish Science, co-financed from EU resources, obtained from the European Regional Development Fund under the Smart Growth Operational Programme, M. Lipowska Grant / Research support from: the TEAM-NET programme of the Foundation for Polish Science, co-financed from EU resources, obtained from the European Regional Development Fund under the Smart Growth Operational Programme.

